# Post-Transcriptional Regulation by Poly(ADP-ribosyl)ation of the RNA-Binding Proteins

**DOI:** 10.3390/ijms140816168

**Published:** 2013-08-05

**Authors:** Yingbiao Ji, Alexei V. Tulin

**Affiliations:** Cancer Biology Program, Fox Chase Cancer Center, Philadelphia, PA 19111, USA; E-Mail: yingbiao.ji@fccc.edu

**Keywords:** *Parp*, *Parg*, poly(ADP-ribose), RNA-binding protein, RNA metabolism

## Abstract

Gene expression is intricately regulated at the post-transcriptional level by RNA-binding proteins (RBPs) via their interactions with pre-messenger RNA (pre-mRNA) and mRNA during development. However, very little is known about the mechanism regulating RBP activities in RNA metabolism. During the past few years, a large body of evidence has suggested that many RBPs, such as heterogeneous nuclear ribonucleoproteins (hnRNPs), undergo post-translational modification through poly(ADP-ribosyl)ation to modulate RNA processing, including splicing, polyadenylation, translation, miRNA biogenesis and rRNA processing. Accordingly, RBP poly(ADP-ribosyl)ation has been shown to be involved in stress responses, stem cell differentiation and retinal morphogenesis. Here, we summarize recent advances in understanding the biological roles of RBP poly(ADP-ribosyl)ation, as controlled by Poly(ADP-ribose) Polymerases (PARPs) and Poly(ADP-ribose) Glycohydrolase (PARG). In addition, we discuss the potential of PARP and PARG inhibitors for the treatment of RBP-related human diseases, including cancer and neurodegenerative disorders.

## 1. Introduction

The regulation of gene expression is not confined to the transcriptional level; it is also tightly regulated at the post-transcriptional level. When a particular RNA transcript is synthesized, there is no guarantee that it will create a functional protein in the cell. To become an active protein, pre-mRNA must be (1) processed into mRNA by alternative splicing and polyadenylation; (2) translocated from the nucleus to the cytoplasm; (3) translated by the protein-synthesizing apparatus; and (4) degraded by the RNA decay machinery [1]. All these RNA processing steps are intricately controlled by RNA-binding proteins (RBPs), which are associated with pre-mRNA/mRNA during the lifespan of a particular RNA [2,3]. Therefore, RBPs play central roles during RNA metabolism [4], and dysregulation of RBP expression can cause a variety of human diseases [5].

**Figure 1:**
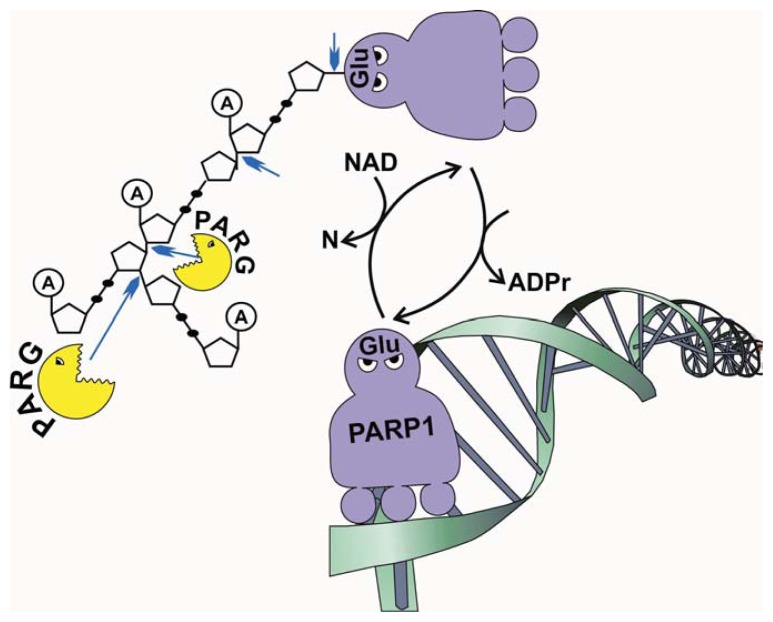
Nuclear Poly(ADP-ribose) turnover. Level of cellular pADPr reflects relative activities of the poly(ADP-ribose) polymerase (PARP) enzyme, which utilizes NAD to create pADPr-modified proteins, and the poly(ADP-Ribose) glycohydrolase (PARG) enzyme, which removes pADPr moieties. Arrowheads indicate cleavage sites of poly(ADP-ribose) by PARG.

RBPs can be modified at the post-translational level by phosphorylation [6–9], ubiquitination [10,11] and poly(ADP-ribosyl)ation (PARylation) [12] to achieve temporal/spatial-specific post-transcriptional control of gene expression. PARylation is performed by Poly(ADP-ribose) polymerases (PARPs), which utilize NAD to synthesize poly(ADP-ribose) polymer (pADPr) with the resulting sizes varying from 2 to 200 ADP-ribose units [13,14] (Figure 1). Poly(ADP-ribosyl)ation alters the physical and enzymatic properties of acceptor proteins which become highly negatively charged [14,15]. However, the resultant level of poly(ADP-ribosyl)ated proteins within a cell depends on the relative activity of PARPs and poly(ADP-ribose) glycohydrolase (PARG), which is responsible for degrading pADPr polymer [16–18] (Figure 1). Therefore, PARylation is a reversible process involved in the regulation of many aspects of biological processes, including chromatin modulation, transcription control and DNA damage repair [12,19,20]. In addition, recent studies have demonstrated that many RBPs are modified by PARylation to regulate post-transcriptional processes. In this review, we highlight the molecular events and developmental processes regulated by RBP PARylation.

## 2. Post-Transcriptional Molecular Events Regulated by PARylation of RBPs

### 2.1. Regulation of Alternative Splicing by PARylation of hnRNPs

**Figure 2:**
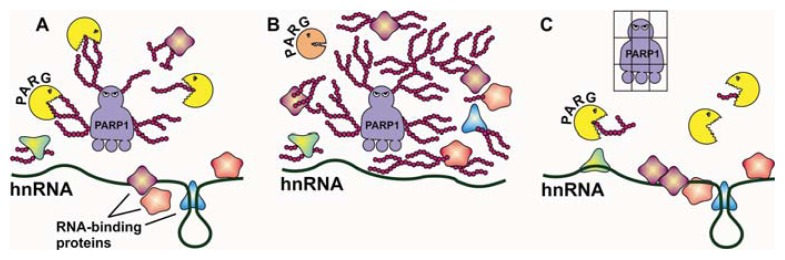
Model explaining the regulatory role of RNA binding proteins (RBP) poly(ADP-ribosyl)ation. (**A**) In a temporal- or spatial-specific way, protein poly(ADP-ribosyl)ation is reversed by PARG activity. Therefore, RBPs bind only partially to poly(ADP-ribose) and will bind to their target heterogeneous nuclear RNAs (hnRNAs) for RNA processing or translational control; (**B**) Once PARG activity is downregulated (high PARP-1–low PARG activities) in different tissues or developmental stages, RBPs are poly(ADP-ribosyl)ated, which inhibits RBPs from binding to hnRNAs. Therefore, the hnRNA transcripts are not processed or translated, and developmental patterns are changed; (**C**) Downregulation of PARP-1 (low PARP-1–high PARG activities) leads to depletion of poly(ADP-ribose). Thus, all RBPs excessively bind to hnRNAs, altering processing and translation.

Heterogeneous nuclear ribonucleoproteins (hnRNPs) are a group of RNA-binding proteins, including at least 20 abundant proteins designated from hnRNP A through U, which mediate the multiple steps of RNA processing [21]. In 1982, it was first reported that several hnRNP proteins with 36, 39 and 42 kDa were associated with mono- or poly(ADP-ribose) in rat liver nuclei using the pulse-chase experiment with [^32^P]-labeled NAD as the substrate [22]. A similar study conducted in 1994 confirmed that hnRNP A1 and A2/B1 are the two major acceptors of mono- or poly(ADP-ribose) in HeLa cells [23]. A proteomic approach further demonstrated that 8 human hnRNP proteins (A1, A2/B1, C1/C2, G, H, K,M and E1) are indeed associated with pADPr through a conserved pADPr-binding domain [24]. The interaction of pADPr with human hnRNPs has been confirmed by different proteomic approaches, such as the use of pADPr-binding macrodomain affinity resin [25], Co-IP with PARP1/2 and PARG antibodies [26] and clickable NAD analogue labeling [27]. So far, a total of 11 human hnRNP proteins ((A1, A2/B1, C1/C2, G, H, K,E1) [24], A3 [28], L [27], M [24,27], U [29]) have been identified to interact with pADPr. It appears that modification of hnRNPs by pADPr is a conserved mechanism between mammals and *Drosophila* because three *Drosophila* hnRNPs (Hrp36, Hrp38 and Hrp40) have been shown to be associated with pADPr *in vivo* [30,31]. Specifically, pADPr binds to a conserved pADPr-binding motif, which is located between RBD-1(RNA-binding domain) and RBD-2 of hnRNP A1 [24]. This 20-amino acid motif belongs to a pADPr-binding consensus sequence identified in DNA damage-related proteins [32]. Mutations of all basic and hydrophobic amino acids into alanine in this motif abolished the ability of hnRNPA2 binding to pADPr *in vitro*, suggesting that this motif is essential for pADPr binding to hnRNPs [24]. RNA electrophoretic mobility shift assay (EMSA) showed that PARylation of hnRNPs inhibits the RNA-binding ability of hnRNPs [31,33,34], suggesting that the biological role of hnRNP PARylation is to modulate the activity of hnRNP proteins. Indeed, experimental evidence has demonstrated that PARylation can regulate at least two hnRNP-dependent post-transcriptional processes, including alternative splicing [31] and translation [33] (Figure 2).

The hnRNP proteins play a primary role in controlling alternative splicing by binding to specific RNA elements [35]. *Drosophila* hnRNPA1 (Hrp38) has been shown to control alternative splicing of the *Ddc* (dopa decarboxylase) gene, which encodes two tissue-specific isoforms [31]. Hrp38 PARylation regulates the splicing pattern of this gene under heat shock treatment [31]. Heat shock treatment can induce PARP1 activity and significantly increase the pADPr level in cells [36]. As a consequence, PARP1 modifies Hrp38 and Hrp40 with pADPr in a noncovalent manner, which causes hnRNPs to dissociate from most transcripts, including the *Ddc* pre-mRNA [31]. It appears that Hrp38 and Hrp40 are the splicing repressors for alternative splicing of exon B in the *Ddc* gene [31]. PARylation of these hnRNPs causes their dissociation from the intronic splicing elements of the *Ddc* pre-mRNA, thus modulating the splicing pathway [31]. Because hnRNP PARylation is controlled by PARP and PARG activities *in vivo* [31], hnRNP PARylation may regulate tissue- or developmental-specific splicing under normal physiological conditions as a consequence of the spatial- and temporal-specific activity of PARP and PARG in the organism (Figure 2).

### 2.2. Inhibition of Phosphorylation of S/R Proteins by Poly(ADP-ribose)

Besides hnRNP proteins, several other splicing factors, including ASF/SF2 [37], SF3B1 [38], SF3A1 and SF3B2 [26], have been found to be associated with pADPr. ASF/SF2, a prototypical serine-arginine-rich protein, is involved in splicing regulation [39]. It has been reported that pADPr binds with ASF/SF2 via either the RRM1 or RS domain, but not with the RRM2 domain [37]. pADPr binding to AS/SF2 inhibited ASF/SF2 phosphorylation by antagonizing the activity of DNA topoisomerase, which acts as a kinase to phosphorylate the serine residues of ASF/SF2 [37]. Because ASF/SF2 phosphorylation can promote splicing [40], pADPr binding to SR proteins may also regulate alternative splicing by modulating phosphorylation of SR protein or by directly influencing the affinity for RNA binding.

### 2.3. Inhibition of Polyadenylation by PARylation of Poly(A) Polymerase

Poly(A) polymerase (PAP) is responsible for adding the poly(A) tail to the mRNA 3′-end after the cleavage of the primary RNA by the 3′-end processing machinery [41]. PARP1 has been found to be one of the components in the 3′-end processing complex by a proteomics study [42]. A recent study further showed that PARP1 can directly PARylate PAR under heat shock stress [43]. Similar to the effect of PARylation on hnRNP activity, PAR PARylation inhibits the RNA-binding ability of PAR, which further reduces the polyadenylation level of target mRNAs [43]. It appears that PAR is mainly modified by activated PARP1 to inhibit mRNA 3′-end processing of the genes whose expression is not induced by heat shock [43]. Therefore, activated PARP1 plays multiple roles to alleviate heat shock stresses, including the induction of heat shock gene expression [36], regulation of splicing [31] and polyadenylation [43] (Figure 2).

### 2.4. Regulation of miRNA-Mediated Gene Silencing by PARylation of the Argonaute Protein Family

Argonaut proteins (Agos) are associated with miRNA and facilitate the ability of miRNAs to execute gene silencing functions by binding to the 3′UTR of target mRNA [44]. Through a study on the roles of PARPs on stress response, Ago2 has been identified to be modified by PARP1 through an RNA-binding domain (PIWI) of Ago2 [45]. In addition, it has been shown that three other Ago proteins (Ago1, Ago3, and Ago4) can be PARylated under nonstress condition [45]. Increasing the level of PARylated Agos in the cells by PARP1 overexpression and PARG knockdown alleviates the inhibitory effect of miRNA on the target genes, suggesting that Ago PARylation is a novel mechanism to regulate miRNA-mediated gene silencing [45].

### 2.5. Controlling Protein Translation and Stability

**Figure 3:**
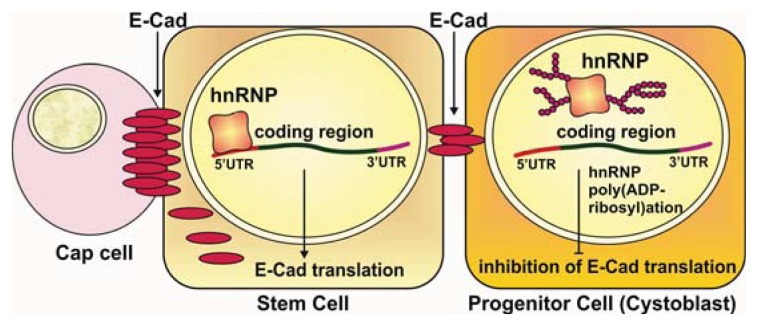
Diagram illustrating how hnRNP poly(ADP-ribosyl)ation controls maintenance of stem cells in the stem cell niche. HnRNP poly(ADP-ribosyl)ation regulates E-cadherin (E-Cad) translation: hnRNP binds to 5′UTR of E-cadherin to promote translation. Once hnRNP is poly(ADP-ribosyl)ated and dissociated from 5′UTR of E-cadherin, its translation is inhibited; Poly(ADP-ribosyl)ation of hnRNP controls germline stem cell maintenance: E-cadherin protein (red) accumulates between stem cell niche cells (Cap Cell) and stem cells, keeping stem cells in the niche. High level of poly(ADP-ribosyl)ation during mitosis and in cystoblasts suppresses translation of E-cadherin. Suppression of E-cadherin production promotes cystoblasts that have not established contacts with cap cells to leave the stem cell niche and differentiate.

Besides splicing control, hnRNPs are also involved in protein translation mediated by cellular Internal Ribosome Entry Site (IRES) elements [33,46]. In *Drosophila*, Hrp38 binds to a G-rich element in the 5′UTR of E-cadherin mRNA to promote its translation, suggesting that Hrp38 is a transacting factor for enhancing IRES-mediated translation [33]. Poly(ADP-ribose) binding to Hrp38 can disrupt the interaction between Hrp38 and the 5′UTR of E-cadherin mRNA, inhibiting its translation [33]. It appears that cells utilize this strategy to modulate the E-cadherin expression level for controlling oocyte localization and self-renewal of germline stem cells during *Drosophila* oogenesis [33] (Figure 3). In mammals, PARylation of Snail (a transcription factor) was able to upregulate the protein stability of Snail to further control E-cadherin expression at the transcriptional level [47]. In addition, it was also found that PARG is associated with Fragile-X mental retardation protein (FMRP), a component of the translational apparatus [48]. These studies suggest that PARylation may be a general mechanism to fine-tune gene expression at the translational level.

### 2.6. Regulation of Ribosomal RNA Processing

It has been observed that more than 40% of PARP1 and pADPr is accumulated in the nucleoli [49,50], the functional center for ribosomal DNA transcription, rRNA processing and assembling [51]. Numerous studies suggested that PARylation also plays active roles in regulating ribosomal DNA (rDNA) transcription [52,53] and ribosomal biogenesis [49,54,55] in the nucleoli. Poly(ADP-ribose) binds to several rRNA-associated nucleolar proteins, including Fibrillarin, Nucleolin and Nucleophosmin in *Drosophila* nucleoli [49]. Indeed, disruption of pADPr metabolism by either PARP1 or PARG loss-of-function causes mislocalization of these proteins and nucleolus fragmentation, which further results in rRNA processing defects [49]. In addition, six yeast nucleolar proteins (UTP7, BUD21, Nob1, Has1, Nop53 and LHP1) have been identified to be PARylated upon expression of human PARP1 in yeast (*S. cerevisiae*), which lacks functional PARP homologues [55]. Therefore, these results suggest that pADPr may serve as a matrix to attract rRNA-binding proteins to the nucleoli for ribosomal biogenesis [49].

### 2.7. Controlling Protein Shuttling to Cajal Body

Cajal bodies (CBs), spherical nuclear suborganelles, are composed of small nuclear ribonucleoproteins (RNP) and their associated RNAs [56]. CBs are the mini-factories for assembly and modification of these RNPs in order to participate in splicing, rRNA processing and telomere maintenance [56]. In *Drosophila*, PARP1 is colocalized with CB resident proteins, such as Colin and Fibrillarin, which are modified by pADPr in the CBs [57]. It appears that PARP1, the essential component of CBs, is responsible for maintaining their structural integrity because PARP1 loss-of-function mutations caused breakage of a single Colin-enriched CB into multiple Colin-containing particles [57]. In contrast, excessive pADPr accumulation in the *Parg* mutant induced the formation of additional CBs in the nucleoplasm by excessive modification of the CB proteins by PARP1 [57]. Interestingly, it was observed that DNA damage, which greatly induces PARP1 activation [14], also disrupted the CB structure and caused CB fragmentation [58]. Based on these results, a model was proposed in which automodified PARP1 acts as the shuttle protein to deliver the protein components to CBs [57].

## 3. The Physiological Processes Mediated by PARylation of the RNA-Binding Proteins

### 3.1. Stress Responses

A number of external stimuli, such as DNA damage, heat shock and arsenate, can potently activate PARP1 to elicit the cellular stress responses. Activated PARP1 interacts with the RNA-binding proteins to regulate several stress-specific physiological processes, including the formation of the larger omega speckles (nuclear stress bodies) [31], the assembly of stress granules (SGs) [29,45] and DNA damage repair [59–61].

#### 3.1.1. Regulation of Omega Speckles in the Nucleus

In *Drosophila*, hnRNPs (hrp36, hrp38 and Hrp40) are associated with a long noncoding RNA (*hnrω-n*) to form omega speckles in the interchromatin space in the nuclei [62]. Heat shock induces the formation of the larger omega speckles with the largest one in the 93D locus where the *hnrω-n* gene is transcribed [62]. It appears that hnRNP PARylation is involved in the formation of the larger omega speckles under heat shock [31]. Heat shock treatment significantly increases the amount of Hrp40 binding to pADPr and causes Hrp40 to redistribute to the 93D locus to form the main omega speckles [31]. Consequently, the failure of the cleavage of PARylated hnRNPs (Hrp38 and Hrp40) in the *Parg* mutant causes much smaller omega speckles to form in the 93D locus after heat shock [31]. Therefore, it was proposed that hnRNP PARylation acts as the shuttle to transport hnRNPs from the active-transcribed chromatin to the 93D locus to form the main omega speckles [31]. Interestingly, the formation of the 93D *hnrω-n* speckles after heat shock is very similar to the nuclear stress bodies composed of the Satellite III transcripts and the splicing factors in human cells [63]. It would be interesting to further investigate the roles of hnRNP PARylation in the formation of the nuclear stress bodies in human cells.

#### 3.1.2. The Assembly of Stress Granules in the Cytoplasm

Another cellular stress response is the formation of stress granules (SG) in the cytoplasm, which is composed of the translationally stalled mRNAs and their associated RNA-binding proteins [64]. SGs serve as the reservoir to store these mRNPs to silence translation during stress processes [65]. It was found that four RNA-binding proteins (Ago2(miRNA-binding protein), G3BP1(RNA-decay factor), TIA-1(translational suppressor) and PABP (poly(A)-binding protein)) were PARylated with pADPr in SGs upon arsenate-induced oxidative stress [45]. Five PARPs (PARP5a,12,13.1,13.2 and PARP15) and two PARGs (PARG99 and PARG102) are also colocalized in SGs [45]. Overexpression of SG-PARPs can induce the formation of SGs without stress, while overexpression of SG-PARG causes the disassembly of SGs in the cells [45]. A recent study further demonstrated that G3BP1 is associated with pADPr in DNA damage-induced stress granules [29]. Therefore, PARylation of the SG-associated RNA-binding proteins is necessary for the assembly of SGs under stress condition [29,45].

#### 3.1.3. DNA Damage Response

PARP1 activation, the earliest response to DNA damage, regulates the chromatin structure and recruits the DNA repair proteins to the DNA-damaged sites [12,66]. For example, ALC1 (amplified in liver cancer 1), a nucleosome-remodeling ATPase, is recruited to the DNA-damaged sites by binding to pADPr, which facilitates relaxing chromatin for DNA repair [67]. In addition, pADPr can also bring the RNA-binding protein NONO to the DNA-damaged sites to determine which pathways the cells should follow to repair the double-stranded DNA breaks (DSBs) [58]. DSBs are repaired by two major pathways, termed nonhomologous end joining (NHEJ) and homologous recombination (HR) [68]. Although NONO, one of the components of spliceosome, is involved in RNA processing and transcription control [69], pADPr generated by activated PARP1 can bind to its RRM1 motif after PARP1 senses DSBs [59]. Recruiting NONO to DSB sites guides the DNA-damage repair machinery to repair DSBs through the NHEJ pathway, rather than the HR pathway [59]. Interestingly, human hnRNP G protein encoded by the *RBMX* gene has been identified as a positive regulator for the HR pathway [60]. HnRNP G is recruited to the DNA-damaged sites in a PARP1-depedent manner [60], which is important for BRAC2 expression [60]. It was also reported that hnRNP U-like 1 (hnRPUL1) is associated and colocalized with PARP1 in DSB sites [61]. Therefore, recruiting the RNA-binding proteins to DSB sites by PARP1 may be a critical step for DNA damage repair.

### 3.2. Regulation of Stem Cell Maintenance and Differentiation

Emerging evidence suggests that PARylation of RBPs also plays important roles in controlling stem cell maintenance and differentiation [33]. In *Drosophila*, E-cadherin expression is upregulated in germline stem cells (GSCs) to anchor stem cells through E-cadherin-mediated adherent junctions in their niche for stem cell maintenance [70]. In addition, elevated E-cadherin is also essential for oocyte localization in the posterior pole of an egg chamber [71]. It has been demonstrated that *Drosophila* hnRNP A1 (Hrp38) controls the translation of E-cadherin for stem cell self-renewal and establishing egg chamber polarity during oogenesis [33]. Hrp38 loss-of-function causes oocyte mislocalization and loss of GSC self-renewal by decreased E-cadherin expression [33]. Mechanically, Hrp38 binds to the 5′UTR of E-cadherin mRNA to control its translation, most likely by an IRES-mediated process [33]. It was further revealed that poly(ADP-ribosyl)ation of Hrp38 inhibits E-cadherin translation in the progenitor cells and promotes stem cell differentiation [33]. Accordingly, *Parg* mutant GSCs lost E-cadherin expression and committed to the fate of progenitor cells for differentiation [33]. Activated PARP1 in the progenitor cells disrupts the interaction of Hrp38 with 5′UTR of E-cadherin mRNA [33]. Therefore, poly(ADP-ribose) accumulation induces the differentiation of the progenitor cells by downregulating Hrp38-dependent E-cadherin translation during *Drosophila* oogenesis [33]. In addition, PARP1 is also required for the induction of pluripotent stem cells (iPSCs) via somatic cell reprogramming [28,72,73]. Interestingly, several RNA-binding proteins (hnRNP A3, splicing factors U2AF 35 kD and 65 kD subunits, U1 snRNP and fragile X mental retardation 1) were also identified to be pADPr-binding proteins in iPSCs [28]. Further studies should be pursued to determine if pADPr binding to these proteins could play significant roles in generating iPSCs.

### 3.3. Regulation of Retinal Morphogenesis

Besides regulating stem cell self-renewal and differentiation, hnRNP PARylation is also involved in regulating eye pattern formation in *Drosophila* [74]. Hrp38 loss-of-function caused a rough-eye phenotype with disorganized ommatidia shown in both the *hrp38* mutants and eye-specific RNAi-knockdown fly [74]. In a similar manner, conditional PARG knockout in the fly eye, which accumulated excessive pADPr, also resulted in a rough eye with disrupted ommatidial structure and reduced photoreceptor cells [74]. Highly expressed E-cadherin mediates the formation of adherent junctions between the membranes of the photoreceptor cells for controlling their patterning and rotation [75]. Overexpression of E-cadherin in *Drosophila* eye can rescue the rough-eye phenotype exhibited in the *hrp38* mutant, while *Parg* mutant eye clones also showed ommatidia orientation defects associated with decreased expression DE-cadherin [74]. Therefore, it appeared that Hrp38 PARylation regulates retinal morphogenesis by controlling E-cadherin expression [74]. This finding supports the previous reports that PARP1 activation in mice caused degeneration of photoreceptor cells during both normal retina development [76] and a mouse model of retinitis pigmentosa [77].

## 4. Interference of pADPr Metabolism to Treat RBP-Related Human Diseases

The mutations of RBP and their cis-binding elements are the underlying causes of a variety of human disorders, including neurodegenerative disease and cancer [5]. Considering that PARylation regulates activities of the pADP-associated RBPs, as outlined above, we proposed that manipulating the pADPr level by PARP and PARG inhibitors could be a potential strategy for treating RBP-related human diseases.

### 4.1. Use of PARP1 Inhibitors to Treat Cancer

PARP1 inhibitors have shown promise in treating BRAC1/2-associated ovarian [78] and prostate cancer [79,80], although the underlying mechanism of action is still controversial [81]. A rationale for inhibiting tumor growth by PARP1 inhibitors is that PARP1 inhibition was demonstrated to cause synthetic lethality by the failure to repair ssDNA breaks [78]. However, since PARP1 also controls chromatin structure to regulate transcription [12], the action of PARP1 inhibitors on tumor cells is likely beyond its role for DNA damage response [81]. As reviewed above, inhibition of PARP1 activity may also have a dramatic effect on RBP-related post-transcriptional processes, providing both a new frontier for cancer treatment and concomitant pitfalls, which should be examined to avoid adverse effects on patients. For example, the finding that PARP1 is required to recruit certain RBPs to DNA damage sites [59–61] implies that PARP1 inhibitors may be used to inhibit the RBP-dependent DNA-repair pathway for cancer treatment. However, further studies should also be pursued to investigate how PARP1 inhibitors change RNA metabolism of cancer cells by influencing RBP activities in the treated tumors.

### 4.2. Use of PARP1 Inhibitors to Treat RBP-Related Neurodegenerative Diseases

HnRNPs, the major pADPr-binding proteins, are involved in the pathogenesis of many neurodegenerative diseases, including Fragile X syndrome [82,83], poly-glutamines (poly-Q) disorders [84,85], Alzheimer’s disease [86] and Amyotrophic Lateral Sclerosis (ALS) [87]. Fragile X syndrome with inherited intellectual disability, including autism, is caused by the inhibition of FMR1 (fragile X mental retardation) gene expression caused by the presence of the triplet (CGG) repeats in the 5′UTR of the FMR1 gene [88]. It has been shown that mouse hnRNP A2B1 binds to the CGG repeats of the FMR1 gene [82,83]. Overexpression of *Drosophila* Hrp36 and Hrp38 (homologues of hnRNP A1) suppressed the CGG-mediated rough-eye phenotype in a *Drosophila* model of Fragile X syndrome, confirming the hypothesis that extensive binding of hnRNPs to the CGG repeats titrates the functions of normal hnRNPs [82,83]. In agreement with this finding, both Hrp36 and Hrp38 loss-of-function caused the rough-eye phenotype [74,84]. In addition, the null mutation of Hrp36 enhanced severity of polyQ-induced neurodegeneration of the eye in the fly model of polyQ diseases [84]. This effect could be alleviated by RNAi knockdown of the noncoding *hsrω-n* RNA, which is associated with Hrp36 [85]. These studies suggest that increased availability of hnRNPs for performing their normal functions should benefit the treatment regimens of hnRNP-related neurodegenerative diseases, such as Fragile X syndrome or polyQ disorders. Therefore, a reduction of hnRNP PARylation by PARP inhibitors, which can increase the amounts of unmodified hnRNPs, may improve treatment for neurodegenerative diseases.

### 4.3. Use of PARG Inhibitors to Treat hnRNP-Dependent Cancer

Based on the fact that inhibition of PARG activity can significantly increase the cellular level of PARylated RBPs, we proposed that PARG inhibitors could be used to block the growth of RBP-dependent tumors. It is well documented that overexpression of hnRNP A1 is correlated with cancer cell proliferation in a variety of tumors, including lung [89], liver [90], colorectal [91,92] and gliomas [93]. Tumor cells prefer to utilize aerobic glycolysis to generate glucose and lactate for their growth, a process known as the Warburg effect [94]. Pyruvate kinase isoform 2 (PKM2) promotes this process and tumorigenesis by interacting with the subunit of HIF-1α (hypoxia-inducible factor-1) and inducing the expression of hypoxia response genes [95]. HnRNP A1 and A2 bind to an intronic splicing silencer to inhibit splicing of an alternative exon to produce the higher ratio of PKM2 to PKM1, an isoform for oxidative phosphorylation, in tumor cells [93]. Therefore, inhibiting the activity of hnRNP A1 to reduce the PKM2 level could be a potential strategy to limit the growth of tumor cells by converting the tumor metabolic state from oxidative phosphorylation to aerobic glycolysis. Because hnRNP PARylation inhibited the RNA-binding activity of hnRNP A1 [31,33], PARG inhibitors may have therapeutic benefit for patients with hnRNP A1-dependent tumors by interfering with the metabolism of cancer cells.

## 5. Summary

In summary, PARylation of the RNA-binding protein often inhibits the RNA-binding activities of RBPs, including hnRNPs, S/R proteins, Poly(A) polymerase and Argonaut proteins, thus regulating these RBP-dependent pathways, such as splicing, polyadeylation, maturation of miRNA and translation. Alternatively, pADPr can also work as the matrix to attract the nucleoli/Cajal Body-specific RBP to these specialized organelles to facilitate rRNA processing and Cajal Body-related functions. Consistent with their roles in molecular events, RBP PARylation controls stress responses and developmental processes, such as stem cell differentiation and retina morphogenesis. Because RBP functions can be regulated by manipulating the activities of PARP and PARG, PARP1 and PARG inhibitors are potential therapeutics to treat a variety of RBP-related cancer and neurodegenerative diseases.
